# Additively Manufactured NiTi and NiTiHf Alloys: Estimating Service Life in High-Temperature Oxidation

**DOI:** 10.3390/ma13092104

**Published:** 2020-05-01

**Authors:** Hediyeh Dabbaghi, Keyvan Safaei, Mohammadreza Nematollahi, Parisa Bayati, Mohammad Elahinia

**Affiliations:** Department of Mechanical, Industrial, and Manufacturing Engineering, The University of Toledo, Toledo, OH 43606, USA; Hediyeh.Dabbaghi@rockets.utoledo.edu (H.D.); Keyvan.SafaeiBaghbaderani@rockets.utoledo.edu (K.S.); mohammadreza.nematollahi@rockets.utoledo.edu (M.N.); Parisa.BayatiMalayeri@rockets.utoledo.edu (P.B.)

**Keywords:** additive manufacturing, high-temperature shape memory alloys, oxidation, NiTiHf, NiTi

## Abstract

In this study, the effect of the addition of Hf on the oxidation behavior of NiTi alloy, which was processed using additive manufacturing and casting, is studied. Thermogravimetric analyses (TGA) were performed at the temperature of 500, 800, and 900 °C to assess the isothermal and dynamic oxidation behavior of the Ni_50.4_Ti_29.6_Hf_20_ at.% alloys for 75 h in dry air. After oxidation, X-ray diffraction, scanning electron microscopy, and energy-dispersive X-ray spectroscopy were used to analyze the oxide scale formed on the surface of the samples during the high-temperature oxidation. Two stages of oxidation were observed for the NiTiHf samples, an increasing oxidation rate during the early stage of oxidation followed by a lower oxidation rate after approximately 10 h. The isothermal oxidation curves were well matched with a logarithmic rate law in the initial stage and then by parabolic rate law for the next stage. The formation of multi-layered oxide was observed for NiTiHf, which consists of Ti oxide, Hf oxide, and NiTiO_3_. For the binary alloys, results show that by increasing the temperature, the oxidation rate increased significantly and fitted with parabolic rate law. Activation energy of 175.25 kJ/mol for additively manufactured (AM) NiTi and 60.634 kJ/mol for AM NiTiHf was obtained.

## 1. Introduction

NiTi is a shape memory alloy (SMA) with unique properties, such as biocompatibility, wear, and corrosion resistance, low modulus of elasticity, and high actuation work output [1,2,3,4]. These characteristics make NiTi useful for functional and smart structures in primary areas of biomedical and aerospace applications. The working temperature of below 100 °C for NiTi limits its functionality and application in the industries that need higher operational temperature. Hence, developing high-temperature shape memory alloys (HTSMAs) has gained attention. Adding the third element to NiTi is an approach for changing the transformation temperatures (TTs). Zr, Hf, Pd, and Au are some of the candidates added to NiTi for altering the TTs and other thermomechanical behaviors. Among all aforementioned alloys, NiTiHf has been shown to be both cost-effective and exhibit better thermomechanical stability. In addition, the composition of NiTiHf is pivotally critical since it can significantly affect the performance and functionality of the part [5,6,7,8]. Recently, with the advancement in additive manufacturing (AM) technology, more complex geometries of NiTi-based alloys are fabricated via methods such as selective laser melting (SLM). Moreover, the ability to tailor the thermomechanical characteristics of SMAs is possible during AM [9,10,11,12,13,14,15,16,17].

One of the well-established characteristics of alloys, when exposed to the atmosphere at various temperatures, especially at high temperatures, is their reaction with oxygen and other gaseous species. This exposure, in turn, will produce metal oxidation on the surface of the alloys, which will cause the alloy to lose its mass to oxidation of the metals present. The oxidation formation also slows down the process of diffusion and mass loss, which can control the permeability of the oxide layers [18,19]. The shape memory effect of HTSMAs usually happens at high temperatures up to 800 °C in some cases. Likewise, additive manufacturing techniques also happen at elevated temperatures, which causes the oxidation of the metal and affects the microstructure [20,21]. Therefore, the study of the oxidation effect is of importance for the optimal performance of NiTi-based alloys. These topics have been studied in other contexts. Chu et al. have worked on the oxidation kinetics of equiatomic NiTi SMA. They observed multilayer oxide layers of an outer TiO_2_ layer, a thin inner TiN_3_ layer, and an intermediate layer of a mixture of TiO_2_ and NiTi [22]. The activation energy of oxidation is another important factor in studying oxidation mechanisms. Basically, activation energy shows the resistance of the material to the oxidation. The activation energy of 226 kJ/mol in this study was obtained from the Arrhenius equation. Firstov et al. investigated the effect of oxidation on NiTi alloys at two specific temperatures of 300 and 800 °C. They found that there are two different oxidation behaviors below and above 500 °C [23]. At higher temperatures, the oxidation kinetics of these alloys obeys the parabolic law, while at a lower temperature, it seems to be well fitted with the linear law. The research on the effect of the addition of the third element on the oxidation kinetics of NiTi has been limited. Lin et al. have studied the influence of Cu addition on the oxidation behavior of TiNi SMAs at 700–1000 °C temperature intervals [24]. The oxidation kinetics of Ti_50_Ni_40_Cu_10_ was well followed by the parabolic rate law at 700–925 °C. An improvement of the oxidation resistance was observed with the addition of Cu when compared to Ti_50.8_Ni_49.2_. However, when compared to Ti_50_Ni_50_ the performance is subpar. The activation energy was calculated to be 180 226 kJ/mol, which was higher than that of Ti_50.8_Ni4_9.2_ but lower than that of Ti_50_Ni_50_. Smialek et al. investigated the effect of Pt addition on the high-temperature oxidation of NiTi alloy at the temperature range of 500–900 °C for 100 h, then compared their results with the NiTi alloy [25,26]. It was found that the mass gain of the binary alloy was twice higher than that of the ternary alloy, which resulted in better oxidation resistance of NiTiPt alloy. The oxidation rate of NiTi was measured to be four times greater than that of NiTiPt alloy, which proved that the Pt addition improved the oxidation resistance of NiTi alloys at high temperatures. The activation energy for both alloys for isothermal oxidation was mostly the same ~250 kJ/mol. Kim et al. [27] studied the oxidation kinetics of Ti–49Ni–12Hf. They reported that the addition of Hf improved the oxidation resistance of NiTi alloy. In the initial stage of oxidation, the oxidation behavior obeyed a parabolic rate law which is followed by a linear law. Five different oxide layers were observed: an outer TiO_2_ layer, a mixed oxide layer of TiO_2_ and NiTiO_3_, a mixture of (Ti, Hf) oxides, and an Ni-rich layer which formed beneath a Hf-rich layer. The existence of the Hf-rich oxide layer formed beneath the outer oxide layer played a significant role in this improvement.

To date, there is no study on the oxidation kinetics of additively manufactured NiTi and NiTiHf. In this work, for the first time, we have evaluated the oxidation kinetics of additively manufactured (AM) NiTi and NiTiHf alloys and compared them with conventionally (CON) made Ni-rich NiTiHf. We have assessed the effects of the oxidation on the characteristics under different exposure conditions. To this end, AM NiTi and NiTiHf were exposed to different temperature environments to measure the oxidization against the conventionally fabricated parts. Based on the results and observations, the activation energy and the mechanism of oxidation are discussed. The results of this study are instrumental in estimating the service life of high-temperature actuators and components made of these functional alloys.

## 2. Materials and Methods

### 2.1. Material Preparation

To produce powder from annealed ${\mathrm{Ni}}_{50.8}{\mathrm{Ti}}_{49.2}$ (at.%) and as-cast ${\mathrm{Ni}}_{50.4}{\mathrm{Ti}}_{29.6}{\mathrm{Hf}}_{20}$ (at.%) ingots, an electrode induction-melting gas atomization (EIGA) technique using TLS Technik GmbH (Bitterfield, Germany) was utilized. Atomization with the inert gas EIGA technique can produce a spherical powder that has low impurity content. In this study, a particle size distribution of 25–75 and 15–63 µm were used for NiTi and NiTiHf powder, respectively, to ensure followability and layer resolution [28,29]. An SLM machine (Phenix Systems PXM, [3D Systems], Rock Hill, SC, USA) equipped with a 300W Ytterbium fiber laser, was employed in this study to fabricate ${\mathrm{Ni}}_{50.8}{\mathrm{Ti}}_{49.2}$ and ${\mathrm{Ni}}_{50.4}{\mathrm{Ti}}_{29.6}{\mathrm{Hf}}_{20}$ parts. To minimize the level of impurity in the fabricated parts, during fabrication the oxygen level of the chamber was held at 800 ppm. 4 × 4 × 10 mm^3^ coupons, which were fabricated directly on a NiTi substrate, were removed from the base plate using electrical discharge machining (EDM). The SLM processing parameters for fabricating NiTi and NiTiHf parts are shown in Table 1. Conventionally fabricated ${\mathrm{Ni}}_{50.8}{\mathrm{Ti}}_{49.2}$ and ${\mathrm{Ni}}_{50.4}{\mathrm{Ti}}_{29.6}{\mathrm{Hf}}_{20}$ samples were cut from the initial ingot and were used for comparison. These ingots were from the same pool that were atomized to make the powder for the SLM fabricated samples. Samples of 40–200 mg were cut and polished from the coupons for thermogravimetric analysis (TGA) measurements. Samples were then ultrasonically cleaned in acetone and dried before the oxidation tests.

### 2.2. Oxidation Test and Characterization Methods

Material oxidation was carried out with a combination of TGA and SDT-Q600 (TA Instruments, New Castle, DE, USA) instrumentation. First, dynamic oxidation tests were performed from room temperature up to 900 °C in order to observe the oxidation start temperatures of AM and conventional NiTiHf and NiTi parts. During these tests, the mass changes of the samples were measured when subjected to the condition of the continual increase in temperature at a constant heating rate of 10 °C/min in air. Next, the isothermal oxidation tests were conducted by maintaining the sample at a constant temperature for a period during which change in mass was recorded. Samples were heated up to the temperatures of 500, 800, and 900 °C in inert nitrogen gas. When the desired temperatures were reached, samples were then oxidized in the air with a flow of 50 mL/min for 75 h for both AM and conventional NiTiHf alloys. The oxidation process continued for 28 h for the binary alloys. For each sample, the mass was constantly measured and recorded over time, and finally, the samples were cooled to room temperature. X-ray diffraction (XRD, Rigaku, Austin, TX, USA) was used to study the oxide phases formed during the isothermal oxidation via a Bruker D8 X-ray diffractometer with Cu-Kα radiation fixed with a diffracted beam monochromatic. Then, samples were mounted in epoxy and polished for microscopy analysis. The final characterization of the specimen after oxidation was performed using a scanning electron microscope (SEM, Thermo Scientific, Waltham, MA, USA) equipped with energy dispersive spectroscopy (EDS).

## 3. Results and Discussion

### 3.1. Oxidation Kinetics

**Figure 1:**
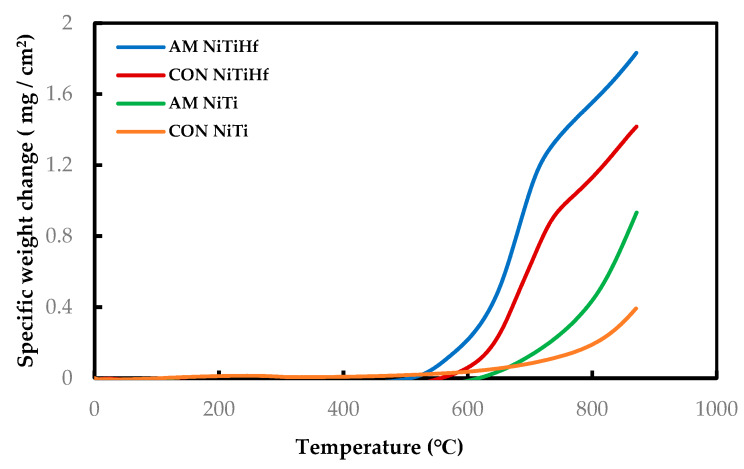
Dynamic thermogravimetric analysis (TGA) measurement of AM and CON NiTi and NiTiHf from 22 to 900 °C in air flow.

Figure 1 displays the dynamic curves of all the samples with and without Hf addition as the oxidation test was performed from room temperature to 900 °C. The results show the development of different oxidation behaviors based on the temperature evolution. Based on the graphs, the oxidation rate is very slow and almost the same for all of the specimens at temperatures lower than 500 °C.

AM NiTiHf begins to oxidize at the lower temperature with respect to others. So that, for both NiTiHf alloys, the oxidation significantly initiates above 500 °C, while the oxidation initiation temperature for NiTi alloys is more than 600 °C.

**Figure 2:**
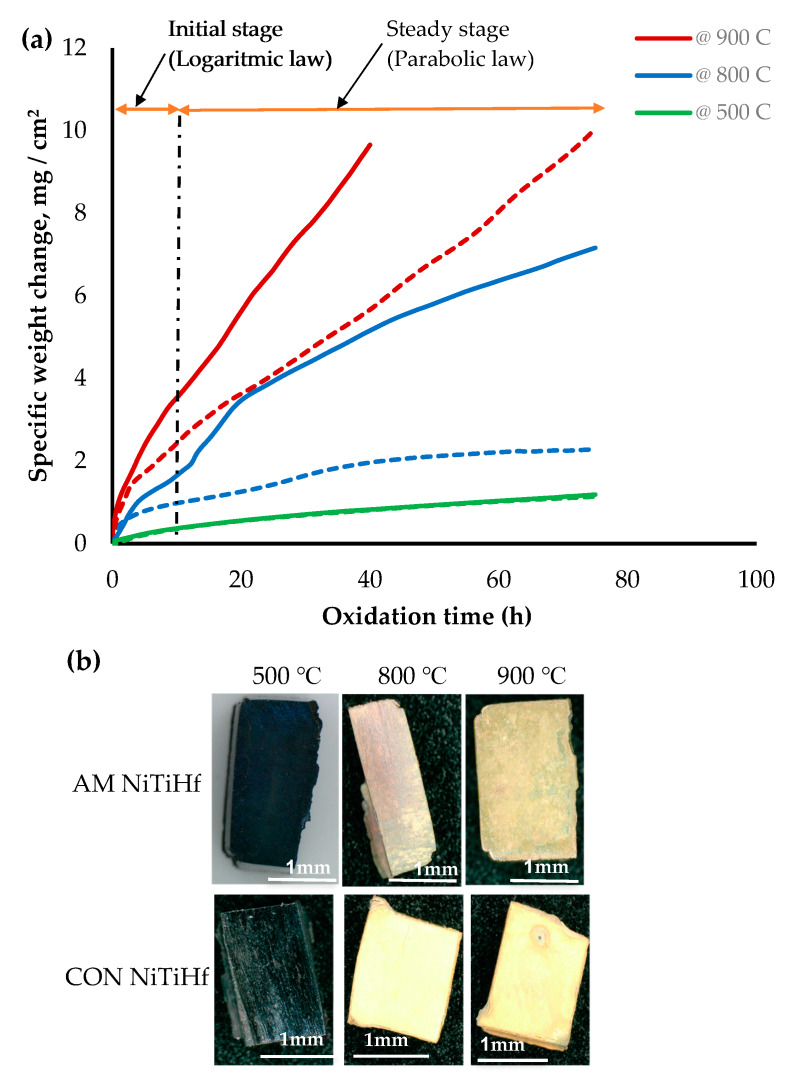
(**a**) Thermogravimetric curves of isothermal oxidation of AM (dash line) and CON (solid line) NiTiHf alloy at 500, 800, and 900 °C; (**b**) Oxidized AM NiTiHf alloys at 500, 800, and 900 °C.

Figure 2 illustrates the oxidation kinetic of AM and CON NiTiHf alloys at different temperatures. The results show that the oxidation rate increased by increasing the temperature. The mass gains for both samples at 500 °C were the same and about less than 1$\mathrm{mg}/{\mathrm{cm}}^{2}$. This indicates that oxidation was not significant at this temperature. At 800 and 900 °C, CON NiTiHf experienced a rapid mass gain against AM NiTiHf, which shows a higher rate of oxidation for CON alloys. For AM samples, the rate of the oxidation at 500 and 800 °C was rather low, while increasing the temperature to 900 °C increased it significantly. This also is evident from the optical images in Figure 2b, which shows the oxidized cross sections for the mentioned samples. Based on the observation, for both AM and CON Hf alloys, the kinetics curves are divided into two stages: the initial stage and steady-state stage.

**Figure 3:**
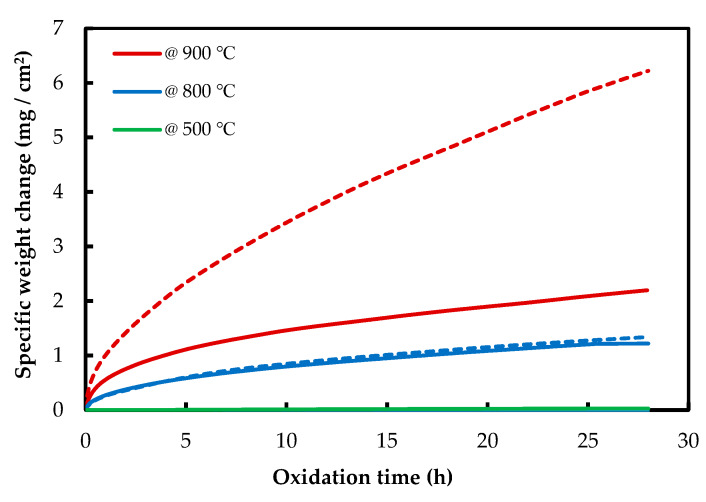
Thermogravimetric curves of isothermal oxidation of AM (dash line) and CON (solid line) NiTi alloy at 500, 800, and 900 °C.

The isothermal oxidation results of AM and CON NiTi alloys are shown in Figure 3. AM NiTi alloys gained more mass than CON alloys, which shows the higher oxidation resistance of CON NiTi alloys. At 500 °C, the mass gain is near zero for both NiTi alloys, which confirms the dynamic TGA results. For AM samples at 500 and 800 °C, the mass gain is rather low, but it increases very fast when it reaches 900 °C. Contrary to NiTiHf alloys, NiTi alloys show a single stage of oxidation, and no initial stage is seen for these alloys.

As is shown in Figure 2 and Figure 3, it can be observed that the mass gain of the AM NiTiHf at 500, 800, and 900 °C is higher than that of AM NiTi alloys, which suggests that the addition of Hf decreases the oxidation resistance of the alloy.

Particular kinetic laws are applied to determine the fluctuation of oxidation rates over time. These values are used to determine an average oxidation rate [30]. The parabolic rate law (Equation (1)) and logarithmic rate law (Equation (2)) are used to analyze the oxidation rate of alloys:

(1) (ΔWA)2=Kpt

(2) (ΔWA)=Log KLt

where ΔW is the mass gain (mg), K is the oxidation constants, A is the unit of the surface area (cm^2^) of the sample, and t (s) is the time of the oxidation. $K_{p}$ can be obtained from the slope of the linear regression line fitted on a (ΔWA)2 versus time plot. The oxidation rate constants and their related correlation coefficient (R) values have been obtained from the linear regression of isothermal oxidation measurement found in Table 2. It is obtained that the oxidation kinetics of both AM and CON NiTi alloys is highly well fitted with the parabolic rate law with a high correlation coefficient of 0.99, while the results for NiTiHf alloys are different. The oxidation kinetics of AM and CON NiTiHf depend on both the time and temperature of oxidation. During the early stage of oxidation (the initial stage), the mass gain curves obey the logarithmic rate law, but after a few hours (the steady-state stage), they follow a parabolic rate law (Figure 2). Due to the small portion of the initial stage in comparison with the steady-state, the $K_{L}$ is negligible, and only $K_{p}$ is reported.

The activation energy ($E_{a})$ obtained from Arrhenius’s equation [31] is a measure of the sensitivity of the oxidation to temperature:

(3) $$k_{r}=A\;e^{-E_{a}/RT}$$

where $k_{r}$ is the rate constant, *A* is a pre-exponential factor, $E_{a}$ is the activation energy, *R* is the gas constant, and *T* is the temperature.

**Figure 4:**
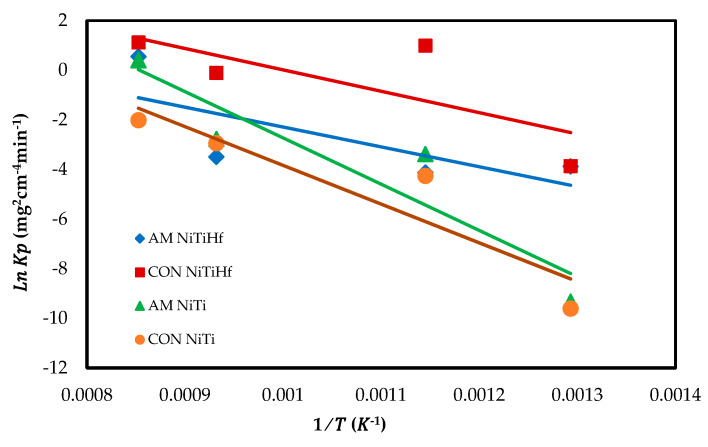
Arrhenius plot of the parabolic rate constant of AM NiTiHf, CON NiTiHf, and AM NiTi alloys.

Figure 4 displays the Arrhenius plot of the oxidation rate constant for all alloys. The activation energy ($E_{a}$) is found to be 60.634 kJ/mol for AM NiTiHf, 91.454 kJ/mol for CON NiTiHf, 175.25 kJ/mol for AM NiTi, and 146.16 kJ/mol for CON NiTi. It can be derived that the addition of Hf decreased the activation energy. The amounts of activation energy confirm the dynamic oxidation result so that the earlier oxidation happened in the alloys with the lower activation energy.

### 3.2. Microstructure Characterization

In the previous section, the oxidation behavior of additively and conventionally manufactured NiTi and NiTiHf alloys was investigated. It was shown that AM and CON NiTi have a single-stage oxidation behavior, while NiTiHf alloys show two-stage oxidation kinetics for both fabrication methods. Moreover, it was discussed that the NiTiHf ternary alloy has less oxidation resistance with respect to NiTi binary alloy. In this section, the possible reasons behind each behavior shown earlier are discussed through the microstructural characterizations.

#### 3.2.1. Microstructural Characterization of NiTiHf Scale

**Figure 5:**
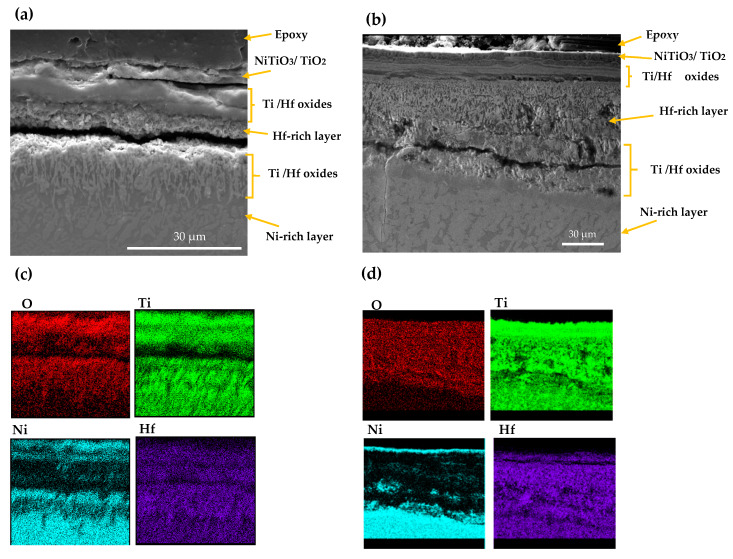
SEM image and X-ray mapping of the cross section of oxidation scale. (**a**,**c**) AM NiTiHf at 800 °C; (**b**,**d**) AM NiTiHf at 900 °C.

The SEM and EDS mappings for the AM NiTiHf sample tested at 900 and 800 °C are shown in Figure 5. In general, outward diffusion of Ti/Hf and inward diffusion of Ni content can be seen using X-ray mapping. Ti cations that have a smaller size than Hf cations can diffuse outward easily [32]. For both samples at 800 and 900 °C, based on the EDS results, the outer layer can be related to the NiTiO_3_/TiO_2_ phase. Lower Gibbs free energy of NiTiO_3_ and TiO_2_, when compared with other Ni/Ti oxides, can explain the oxidation formation of the first layer [33,34]. The next layer shows a Ni depleted layer containing TiO_2_ and HfO_2_ oxides. After the Ti/Hf oxide layer, a high concentration Hf content layer, a Hf-rich layer, with a needle-like structure can be seen at both temperatures. By passing the Hf-rich layer, a Ni-rich segment with around 70 at.% of Ni content can be seen for AM NiTiHf at 800 °C, while at 900 °C, an interlayer of Ti/Hf oxide has been formed between Hf-rich and Ni-rich layers. The significantly higher oxidation rate of AM NiTiHf at 900 °C in comparison with 800 °C can, therefore, be explained by the role of the Hf-rich layer. At 900 °C, the thickness of the Hf-rich layer is about 50% of the total width of the oxide film, while this layer thickness is around 25% for 800 °C. It can, therefore, be seen that the Hf-rich layer plays a vital role in the weight gain and oxidation kinetic behavior of the parts. The Hf-rich oxide layer could also play a role as a barrier for Ti cation and can block the outward diffusion of Ti in the oxide scale. The layer does not, however, stop oxygen diffusion [31,35]. Gradually, the Hf-rich layer becomes the main mechanism of oxidation for AM NiTiHf. As shown in the SEM images, long cracks have been formed along with the oxide layers. These cracks can explain the change of the oxidation kinetic law from logarithmic to parabolic for the AM NiTiHf samples (Figure 2). The sudden change in the oxidation behavior indicates that the crack formation is not the result of the shrinkage that happened during the cooling stage.

**Figure 6:**
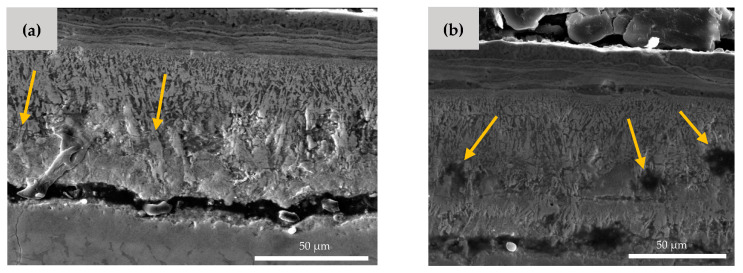
SEM image of AM NiTiHf at 900 °C. (**a**) Microcrack and (**b**) micro void formation in the Hf-rich oxide layer.

As shown in Figure 6, there are also some microcracks and micropores formed in the Hf-rich oxide layer. The microcrack formation is a result of the brittleness of the HfO_2_ phase [27]. The small pores, which are highlighted by arrows in Figure 6b, can be explained by the Kirkendall effect [36,37]. The different diffusion speed of Hf, Ni, and O element results in the formation of the vacant areas and porosities inside the material. It is worth noting that these microcracks and pores facilitate the entrance of oxygen, which can cause more mass gain in the NiTiHf samples in comparison to the NiTi samples.

**Figure 7:**
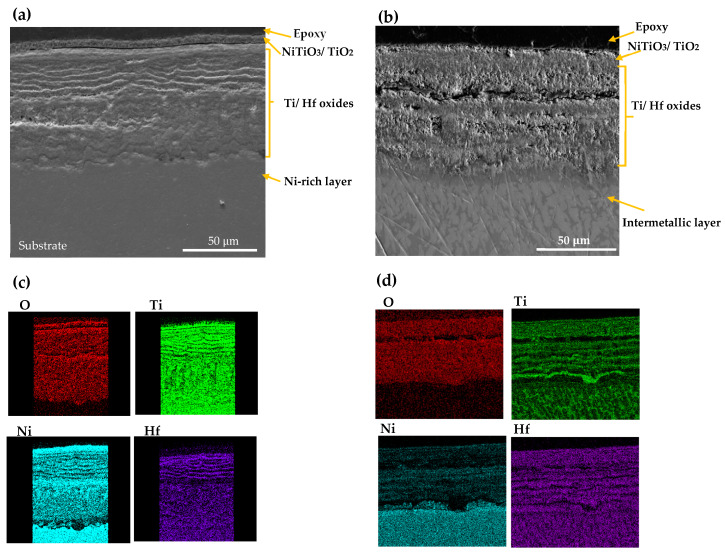
SEM image and X-ray mapping of the cross section of oxidation scale. (**a**,**c**) AM NiTiHf at 800 °C; (**b**,**d**) AM NiTiHf at 900 °C.

SEM morphology and EDS mappings analysis of CON NiTiHf 800 and 900 °C samples are shown in Figure 7. The overall scale morphology is almost identical for both the AM and CON manufacturing process. Like AM samples, due to the higher oxidation temperature, the amount of inward diffusion of the Ni cation for the sample at 900 °C is more than the sample that was tested at 800 °C. However, CON NiTiHf samples showed lower oxidation resistance in comparison to AM NiTiHf samples. The surface roughness could be a possible source of difference between AM and CON samples. The higher surface roughness of the as-fabricated AM samples with respect to the conventionally fabricated parts could increase the surface area in contact with oxygen and result in a higher oxidation rate. However, in this study, difference in the surface roughness is not the case, since all the samples (AM and CON) were polished entirely before performing the TGA tests. Grain size could be another possible explanation for the different oxidation behavior of the AM and CON samples. Based on the study by Li et al. [38], samples with more extended grain boundaries have lower oxidation resistance in comparison to samples with shorter grain sizes. In this study, CON samples are as-cast without any post-treatment, which results in a large grain size in comparison with AM samples, which have a small grain size due to the high cooling rate. Moreover, during the fabrication of AM parts due to the high energy density of the laser, Ni evaporation could occur. Different alloy compositions could be a possible source of variation in the oxidation rate of AM and CON NiTiHf. In a previous study by our team [28], it was shown that for samples fabricated with high energy density, Ni evaporation results in composition change to Ti-rich alloys. As a result, CON and AM parts do not have the same composition, which could be another explanation of the different oxidation kinetics of these samples, which needs further investigation.

**Figure 8:**
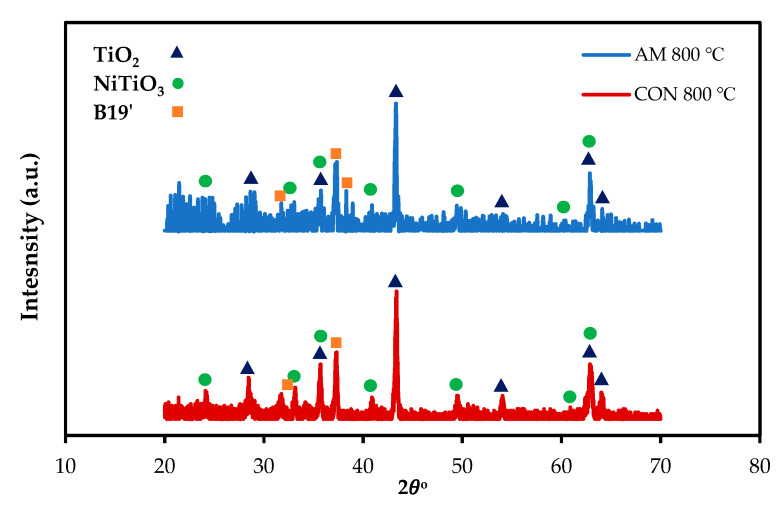
X-ray diffraction patterns of the surface oxide scales on the AM and CON NiTiHf alloys after oxidation at 800 and 900 °C.

To confirm the EDS result, parallel-beam geometry XRD with a low angle of the incidence was performed for AM and CON NiTiHf alloys at 800 °C (Figure 8). For both alloys, the TiO_2_ rutile and NiTiO_3_ are the main phases of the scale surfaces. The XRD analysis confirms the microstructural characterizations.

#### 3.2.2. Microstructural Characterization of NiTi Scale

**Figure 9:**
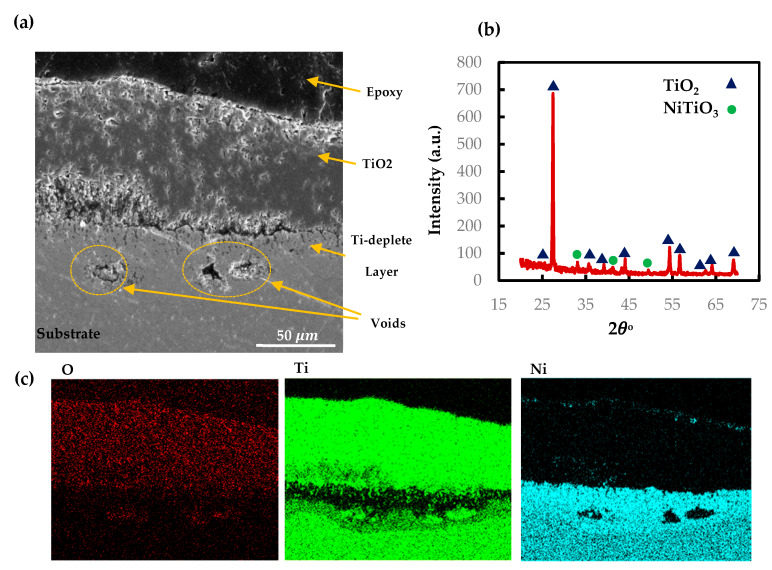
(**a**) SEM micrographs of cross sections of AM NiTi alloy at 900 °C, (**b**) XRD patterns performed on the surface of the oxidized AM NiTi at 900 °C, (**c**) X-ray mapping of the cross section of the oxide layer of AM NiTi.

As discussed in Section 3.1, the oxidation kinetics of AM and CON NiTi follow the single-stage parabolic law. To show the microstructure of the oxide scale for the AM NiTi part at 900 °C, SEM, X-ray mapping, and XRD of the oxide cross section are presented in Figure 9. The SEM shows a dense oxide layer adhered properly to the parts. The X-ray mapping suggests a Ti oxide layer at the outer surface with a thickness of 75 μm on average. The SEM results are also confirmed by the low-angle parallel beam XRD pattern showing the TiO_2_ rutile phase at the outermost layer with a very small portion of NiTiO_3_ phase. Similar to the NiTiHf cases, the reason for TiO_2_ oxide formation can be explained based on Gibbs free energy [39]. The X-ray mapping shows the outward diffusion of Ti to react with oxygen atoms migrate inward through the part. However, the Ti diffusion is faster than the oxygen toward the TiO_2_ rutile layer [40]. Thus, the rutile grows outward, while Ni diffuses inward and forms a Ni-rich layer beneath the rutile oxide. The exothermic nature of oxide formation leads to increasing the thickness of the oxide layer by increasing the temperature or time, and therefore, as time passes, the oxide layer adds up. The parabolic kinetic law of NiTi oxidation behavior can be explained by the barrier effect of rutile on Ti outward migration. Ti diffusion through the rutile layer is inversely proportional to the rutile thickness so that by increasing the oxide thickness, the speed of Ti diffusion drops. The void formation inside the rutile layer closing the Ni-rich interface might be the result of the rutile crystals growing in different directions. The dominant direction of the rutile growth at the initial stage is outward, but as the oxide layer increases, the rutile crystals tend to grow in the lateral direction. As a result, the different growth rate and direction between the rutile crystals creates micro voids, mostly near the Ni-rich interface. There are also some voids in the Ti-deplete region, which are encircled (Figure 9a). As discussed before regarding the difference between the diffusion rate of rutile and oxygen atoms, the Kirkendall effect happens in matter with a higher diffusion rate [41,42]. The other defect happening during the oxidation process is a long crack forming along the TiO_2_ and Ti-deplete interface. We believe that this crack happened during the cooling stage and not during the oxidation process. The brittleness of the TiO_2_ oxide layer and different thermal expansion of the two layers causes crack formation in the cooling stage. The oxidation kinetics of parabolic law with no deviation during the process confirms no cracking happened during the heating.

For NiTi alloys, the AM process decreases the oxidation resistance of the alloy compared to the conventionally fabricated NiTi samples. As discussed earlier, the high temperature of the SLM process resulting in Ni loss during fabrication makes the alloy richer in Ti. It is well reported that the richer Ti is, the more oxidation happens [22,27,43,44]. As reported in our previous works [28,45,46,47,48] different SLM process parameters or post processing result in different compositions, microstructures, and thermomechanical behaviors of the as-fabricated NiTi alloys. Such a variation in composition and microstructure of as-fabricated alloys can differently affect the oxidation resistance. For example, there is some literature on the effect of porosity on oxidation behavior; however, we did not take this into account since the measured density was more than 98% and the samples were polished before the TGA test, and it was expected that all the pores were removed on the surface of both AM and CON samples. As a result, in our case, porosity could not possibly be considered as an effective factor in the oxidation behavior. However, it has been shown in the literature that the open porosity on the surface formed during SLM fabrication, or in case of insufficient polishing, can play a role as a canal for the oxidation entrance and can increase the mass gain of the parts due to the increase of exposed surface [49,50].

## 4. Conclusions

The high-temperature oxidation behavior of additively and conventionally fabricated Ni_50.4_Ti_29.6_Hf_20_ and Ni_50.8_Ti were investigated. The dynamic TGA results showed that oxidation starts above 500 °C for both alloys. NiTiHf alloys in both manufacturing methods showed lower activation energy in comparison with NiTi alloys. The AM process caused lower activation energy for NiTi alloys, while this trend is the opposite for NiTiHf. Both AM and CON NiTiHf alloys followed the logarithmic law at the initial stage of isothermal oxidation kinetics followed by the parabolic law, which shows a higher oxidation rate at the beginning of oxidation for NiTiHf alloys. On the other hand, the oxidation kinetics of NiTi alloys showed a single stage of parabolic behavior. For both NiTi and NiTiHf, as the oxidation temperature increased, the oxidation rate followed in trend. NiTi alloys also showed higher oxidation resistance in comparison with NiTiHf alloys. The microstructure characterization, including XRD, SEM, and EDS analysis confirmed different oxidation layer morphology for AM and CON NiTiHf. For AM NiTiHf alloys, it was shown that Hf-rich layer formation plays a significant role in oxidation kinetics. In addition, it was discussed that crack formation upon the heating stage of oxidation caused the two-stage oxidation behavior for NiTiHf. For AM NiTi, the oxidation layer consisted of a compact TiO_2_ layer, which confirmed the single-stage oxidation kinetics. It was shown that the additive manufacturing process increased the oxidation resistance of NiTiHf alloy, while this trend is the opposite for NiTi alloy. The different sets of AM process parameters including laser power, scanning speed, and hatch spacing can result in various microstructures and compositions. The significant difference between the energy densities employed for fabricating NiTi (83.3 $\frac{J}{{\mathrm{mm}}^{3}}$ ) and NiTiHf (313 $\frac{J}{{\mathrm{mm}}^{3}})$ could be a source of the different trends in the oxidation behavior of both alloys. However, understanding the effect of the AM process parameters on the oxidation behavior needs further investigation. To understand the effect of the AM process on the oxidation behavior of NiTi(Hf) alloys, AM parts with various sets of process parameters and a wide range of energy density need to be characterized. Moreover, further studies on isothermal oxidation with short durations are needed to understand the mechanism of transition from the initial stage to steady-state for NiTiHf alloy.

## Figures and Tables

**Table 1:** The processing parameters employed during selective laser melting (SLM) fabrication.

Material	Laser Power (P, W)	Scanning Speed (v, mm/s)	Hatch Spacing (h μm)	Layer Thickness (t μm)	Energy Input (E $\frac{J}{mm^{3}}$)
AM NiTi	250	1250	80	30	83.3
AM NiTiHf	150	200	80	30	313

**Table 2:** Calculated parabolic rate constants for AM and CON NiTiHf and AM NiTi. $K_{p}$ unit is $\left({\mathrm{mg}}^{2}\;{\mathrm{cm}}^{-4}\;h^{-1}\right)$.

Alloys	Constants	Temperature (°C)			
	$K_{p\;}\left({\mathrm{mg}}^{2}{\mathrm{cm}}^{-4}h^{-1}\right)$ R	500	700	800	900
AM NiTiHf	$K_{p\;}$	0.0208	0.0162	0.0305	1.739
	R	0.99	0.99	0.99	0.99
CON NiTiHf	$K_{p\;}$	0.0210	2.702	0.9	3.07
	R	0.99	0.99	0.99	0.99
AM NiTi	$K_{p\;}$	0.000089	0.0341	0.0632	1.5
	R	0.99	0.99	0.99	0.99
CON NiTi	$K_{p\;}$	0.000067	0.0144	0.0529	0.1339
	R	0.99	0.99	0.99	0.99

## References

[1] Mohajeri M., Case R., Haghgouyan B., Lagoudas D.C., Castaneda H. (2020). Loading influence on the corrosion assessment during stress-induced martensite reorientation in nickel-titanium SMA. Smart Mater. Struct..

[2] Nematollahi M., Mehrabi R., Callejas M.A., Elahinia H., Elahinia M. A two-way architectural actuator using NiTi SE wire and SME spring. Proceedings of the Active and Passive Smart Structures and Integrated Systems XII.

[3] Nematollahi M., Baghbaderani K.S., Amerinatanzi A., Zamanian H., Elahinia M. (2019). Application of NiTi in Assistive and Rehabilitation Devices: A Review. Bioengineering.

[4] Young B., Haghgouyan B., Lagoudas D.C., Karaman I. (2019). Effect of Temperature on the Fracture Toughness of a NiTiHf High Temperature Shape Memory Alloy. Shape Mem. Superelasticity.

[5] Ma J., Karaman I., Noebe R.D. (2010). High temperature shape memory alloys. Int. Mater. Rev..

[6] Benafan O., Gaydosh D.J. (2017). High temperature shape memory alloy Ni50.3Ti29.7Hf20torque tube actuators. Smart Mater. Struct..

[7] Jahadakbar A., Nematollahi M., Safaei K., Bayati P., Giri G., Dabbaghi H., Dean D., Elahinia M. (2020). Design, Modeling, Additive Manufacturing, and Polishing of Stiffness-Modulated Porous Nitinol Bone Fixation Plates Followed by Thermomechanical and Composition Analysis. Metals.

[8] Haghgouyan B., Young B., Karaman I., Lagoudas D.C. Fracture toughness of martensitic NiTiHf high-temperature shape memory alloy. Proceedings of the Behavior and Mechanics of Multifunctional Materials XIII.

[9] Elahinia M., Moghaddam N.S., Andani M.T., Amerinatanzi A., Bimber B.A., Hamilton R.F. (2016). Fabrication of NiTi through additive manufacturing: A review. Prog. Mater. Sci..

[10] Saghaian S.S.E., Moghaddam N.S., Nematollahi M., Saedi S., Elahinia M., Karaca H.E. (2018). Mechanical and shape memory properties of triply periodic minimal surface (TPMS) NiTi structures fabricated by selective laser melting. Boil. Eng. Med..

[11] Dadbakhsh S., Speirs M., Kruth J.-P., Schrooten J., Luyten J., Van Humbeeck J. (2014). Effect of SLM Parameters on Transformation Temperatures of Shape Memory Nickel Titanium Parts. Adv. Eng. Mater..

[12] Ma J., Franco B., Tapia G., Karayagiz K., Johnson L., Liu J., Arroyave R., Karaman I., Elwany A. (2017). Spatial Control of Functional Response in 4D-Printed Active Metallic Structures. Sci. Rep..

[13] Bormann T., Schumacher R., Müller B., Mertmann M., De Wild M. (2012). Tailoring Selective Laser Melting Process Parameters for NiTi Implants. J. Mater. Eng. Perform..

[14] Haberland C., Elahinia M., Walker J.M., Meier H., Frenzel J. (2014). On the development of high quality NiTi shape memory and pseudoelastic parts by additive manufacturing. Smart Mater. Struct..

[15] Mehrpouya M., Gisario A., Rahimzadeh A., Nematollahi M., Baghbaderani K.S., Elahinia M. (2019). A prediction model for finding the optimal laser parameters in additive manufacturing of NiTi shape memory alloy. Int. J. Adv. Manuf. Technol..

[16] Ghayoor M., Lee K., He Y., Chang C.-H., Paul B.K., Pasebani S. (2020). Selective laser melting of 304L stainless steel: Role of volumetric energy density on the microstructure, texture and mechanical properties. Addit. Manuf..

[17] Nematollahi M., Jahadakbar A., Mahtabi M.J., Elahinia M., Namatollahi M. (2019). Additive manufacturing (AM). Met. Biomed. Devices.

[18] Nishida K., Narita T. (1988). Introduction to High Temperature Oxidation of Metals.

[19] Ghayoor M., Mirzababaei S., Lee K., He Y., Chang C., Paul B.K., Pasebani S. (2019). Strengthening of 304L Stainless Steel by Addition of Yttrium Oxide and Grain Refinement during Selective Laser Melting.

[20] Nordin N.A.B., Bin Johar M.A., Bin Ibrahim M.H.I., Bin Marwah O.M.F. (2017). Advances in High Temperature Materials for Additive Manufacturing. IOP Conf. Series Mater. Sci. Eng..

[21] Ghayoor M., Lee K., He Y., Chang C.-H., Paul B.K., Pasebani S. (2019). Microstructural Analysis of Additively Manufactured 304L Stainless Steel Oxide Dispersion Strengthened Alloy. Microsc. Microanal..

[22] Chu C., Wu S.-K., Yen Y. (1996). Oxidation behavior of equiatomic TiNi alloy in high temperature air environment. Mater. Sci. Eng. A.

[23] Firstov G., Vitchev R., Kumar K.H., Blanpain B., Van Humbeeck J. (2002). Surface oxidation of NiTi shape memory alloy. Biomaterials.

[24] Lin K.-N., Wu S.-K. (2009). Oxidation Behavior of Ti 50 Ni 40 Cu 10 Shape-Memory Alloy in 700–1000 °C Air. Oxid. Met..

[25] Smialek J.L., Humphrey D.L., Noebe R.D. (2007). Oxidation Kinetics of a NiPtTi High Temperature Shape Memory Alloy.

[26] Smialek J.L., Humphrey D.L., Noebe R.D. (2010). Comparative Oxidation Kinetics of a NiPtTi High Temperature Shape Memory Alloy. Oxid. Met..

[27] Kim K.M., Yeom J.T., Lee H.-S., Yoon S.-Y., Kim J.H. (2014). High temperature oxidation behavior of Ti–Ni–Hf shape memory alloy. Thermochim. Acta.

[28] Nematollahi M., Toker G., Saghaian S.E., Salazar J., Mahtabi M., Benafan O., Karaca H., Elahinia M. (2019). Additive Manufacturing of Ni-Rich NiTiHf 20: Manufacturability, Composition, Density, and Transformation Behavior. Shape Mem. Superelasticity.

[29] Toker G.P., Nematollahi M., Saghaian S.E., Baghbaderani K.S., Benafan O., Elahinia M., Karaca H.E. (2020). Shape memory behavior of NiTiHf alloys fabricated by selective laser melting. Scr. Mater..

[30] Khanna A.S. (2002). Introduction to High Temperature Oxidation and Corrosion.

[31] Atkins P., de Paula J. (2011). Physical Chemistry for the Life Sciences.

[32] Zhao X., Xu J., Tang L., Gong S. (2007). High temperature oxidation behavior of NiTiNb intermetallic alloys. Intermetallics.

[33] Tsao T.-K., Yeh A.-C., Kuo C.-M., Murakami H. (2016). High Temperature Oxidation and Corrosion Properties of High Entropy Superalloys. Entropy.

[34] Dagdelen F., Ercan E. (2013). The surface oxidation behavior of Ni–45.16%Ti shape memory alloys at different temperatures. J. Therm. Anal. Calorim..

[35] Atkins P., de Paula J., Keeler J. (2018). Atkins’ Physical Chemistry.

[36] He B., Xu G., Zhou M., Yuan Q. (2016). Effect of Oxidation Temperature on the Oxidation Process of Silicon-Containing Steel. Metals.

[37] Shabalovskaya S., Anderegg J., Laab F., Thiel P.A., Rondelli G. (2003). Surface conditions of Nitinol wires, tubing, and as-cast alloys. The effect of chemical etching, aging in boiling water, and heat treatment. J. Biomed. Mater. Res..

[38] Li L., Gong X., Ye X., Teng J., Nie Y., Li Y., Lei Q. (2018). Influence of Building Direction on the Oxidation Behavior of Inconel 718 Alloy Fabricated by Additive Manufacture of Electron Beam Melting. Materials.

[39] Hirnyj S.I. (2012). Thermochemistry of Oxide Films Formed on Zr-Based Metallic Glasses.

[40] Kekare S.A., Shelton D.K., Aswath P.B. (1993). Oxidation of High-Temperature Intermetallics.

[41] Kirkendall E.O. (1942). Diffusion of zinc in alpha brass. Trans. AIME.

[42] Nakajima H. (1997). The discovery and acceptance of the Kirkendall Effect: The result of a short research career. JOM.

[43] Xu C.H., Ma X.Q., Shi S.Q., Woo C.H. (2004). Oxidation behavior of TiNi shape memory alloy at 450–750 °C. Mater. Sci. Eng. A.

[44] Smialek J.L., Garg A., Rogers R.B., Noebe R.D. (2012). Oxide Scales Formed on NiTi and NiPtTi Shape Memory Alloys. Met. Mater. Trans. A.

[45] Farjam N., Nematollahi M., Andani M.T., Mahtabi M.J., Elahinia M. (2020). Effects of size and geometry on the thermomechanical properties of additively manufactured NiTi shape memory alloy. Int. J. Adv. Manuf. Technol..

[46] Moghaddam N.S., Saedi S., Amerinatanzi A., Hinojos A., Ramazani A., Kundin J., Mills M.J., Karaca H.E., Elahinia M. (2019). Achieving superelasticity in additively manufactured NiTi in compression without post-process heat treatment. Sci. Rep..

[47] Saedi S., Moghaddam N.S., Amerinatanzi A., Elahinia M., Karaca H.E. (2018). On the effects of selective laser melting process parameters on microstructure and thermomechanical response of Ni-rich NiTi. Acta Mater..

[48] Biffi C.A., Bassani P., Nematollahi M., Moghaddam N.S., Amerinatanzi A., Mahtabi M., Elahinia M., Tuissi A. (2019). Effect of Ultrasonic Nanocrystal Surface Modification on the Microstructure and Martensitic Transformation of Selective Laser Melted Nitinol. Materials.

[49] Jia Q., Gu D. (2014). Selective laser melting additive manufactured Inconel 718 superalloy parts: High-temperature oxidation property and its mechanisms. Opt. Laser Technol..

[50] Kong D., Dong C., Ni X., Li X. (2019). Corrosion of metallic materials fabricated by selective laser melting. NPJ Mater. Degrad..

